# *Candida Auris*, An Agent of Hospital-Associated Outbreaks: Which Challenging Issues Do We Need to Have in Mind?

**DOI:** 10.3390/microorganisms8020181

**Published:** 2020-01-28

**Authors:** Raquel Sabino, Cristina Veríssimo, Álvaro Ayres Pereira, Francisco Antunes

**Affiliations:** 1 Reference Unit for Parasitic and Fungal Infections, Infectious Diseases Department, National Institute of Health Dr. Ricardo Jorge. Avenida Padre Cruz, 1649-016 Lisbon, Portugal; cristina.verissimo@insa.min-saude.pt; 2 Instituto de Saúde Ambiental, Faculdade de Medicina da Universidade de Lisboa, Av. Prof. Egas Moniz, Ed. Egas Moniz, 1649-028 Lisboa, Portugal; alvaro.pereira@chln.min-saude.pt (Á.A.P.); fantunes@medicina.ulisboa.pt (F.A.); 3 Serviço de Doenças Infeciosas, Centro Hospitalar Universitário Lisboa Norte/Hospital de Santa Maria, Av. Prof. Egas Moniz, 1649-028, Lisboa, Portugal

**Keywords:** *Candida auris*, hospital infection, environmental contamination, horizontal transmission, azole resistance

## Abstract

The emergence of *Candida auris* is considered as one of the most serious problems associated with nosocomial transmission and with infection control practices in hospital environment. This multidrug resistant species is rapidly spreading worldwide, with several described outbreaks. Until now, this species has been isolated from different hospital surfaces, where it can survive for long periods. There are multiple unanswered questions regarding *C. auris*, such as prevalence in population, environmental contamination, effectiveness of infection prevention and control, and impact on patient mortality. In order to understand how it spreads and discover possible reservoirs, it is essential to know the ecology, natural environment, and distribution of this species. It is also important to explore possible reasons to this recent emergence, namely the environmental presence of azoles or the possible effect of climate change on this sudden emergence. This review aims to discuss some of the most challenging issues that we need to have in mind in the management of *C. auris* and to raise the awareness to its presence in specific indoor environments as hospital settings.

## 1. Introduction

Infections caused by yeasts belonging to *Candida* genus are classified as candidiasis (includes candidemia). From 150 *Candida* species described, some are a part of our normal microbiota and only 10% are known to be responsible for infections in humans, being *C. albicans*, the most frequent etiological agent [1].

Several *Candida* species are known to be pathogenic in healthy individuals, affecting skin and mucous membranes. However, over the last two decades, invasive candidiasis is emerging in critically ill patients, due to several factors such as aging populations with many comorbidities, extended stays in hospital wards, diabetes, use of invasive devices, broad-spectrum antibiotics, extensive surgeries, stem cell transplantation, and immunosuppression (AIDS, and immunosuppressive and immunomodulatory therapy) [2].

*Candida* infections are ranked as the most common of nosocomial infections caused by fungi, emerging as a serious public health problem worldwide [3].

Invasive candidiasis may be caused by more than 150 *Candida* species [3]. *C. albicans* is the most frequent species causing invasive candidiasis in human patients, however, in the last decade, a wide range of non-*albicans* species have progressively been rising as emergent human pathogens, mainly *C. glabrata*, *C. parapsilosis*, and *C. tropicalis* and *Pichia kudriavzevii* (former *C. krusei*) [2,3]. One of the main reasons for this rising is the introduction of fluconazole prophylaxis/therapy, which led to the positive selection of azole-less susceptible/resistant species [4].

*C. auris* was first described in 2009, after being isolated from the external ear canal of an inpatient in a Japanese hospital [5]. Since then, reports of *C. auris* infections, including fungemia, wound infections and otitis, have been most commonly identified [6]. Until now, this species has been associated with hospital environment, being found in several different hospital surfaces, where it can survive for long periods. This multidrug resistant species is rapidly spreading worldwide, with several described outbreaks. One of those was found to be linked to reusable axillary temperature probes [7]. Commercially available biochemical-based tests, used in laboratories to identify fungi, cannot differentiate *C. auris* from some other yeasts species, if there was no update of the associated database [8]. Hence, *C. auris* is considered as one of the most serious emerging pathogen, with severe public health implications. This review aims to discuss some of the most challenging issues that should be the focus in the management of *C. auris*.

## 2. What Is Already Known About This Topic?

### 2.1. Which Specific Conditions Can Explain the Recent Increase of Candida Infections and the Recent Emergence of C. auris?

In recent years, *C. auris* has emerged simultaneously in different countries as a new species able to colonize and infect patients, causing death in a high proportion of these last ones.

Until 2009, only four isolates were retrospectively identified as *C. auris*. Three of these isolates were initially identified as *C. haemulonii* and one was not identified to species level [9,10].

In fact, with the purpose to investigate whether *C. auris* had emerged in recent times or if it had been misidentified in the past, an extensive investigation was conducted within the pool of uncommon *Candida* spp. included in the SENTRY global fungal collection (15,271 isolates of *Candida* spp. collected during 2004–2015 from Asia, Europe, Latin America, and North America) [11]. This study identified a single *C. auris* isolate from Pakistan dating back to 2008, which had not been previously recognized. In 2011, the first three cases of bloodstream fungemia caused by *C. auris* were reported, highlighting antifungal resistance and the ability to cause invasive infections and from those, one was obtained from a microbiological sample collected in 1996 as invasive fungal infection isolate [6]. There are no other unidentified *C. auris* strains prior to 1996 [9].

The US Centers for Disease Control and Prevention (CDC) reviews of other large-scale isolate collections also confirmed the finding that *C. auris*, as well as *C. haemulonii*, were relatively rare before 2009. These findings suggest that *C. auris* emerged as a cause of human infections primarily in the last decade [10].

This global emergence has been attributed to the near-simultaneous appearance in five geographically restricted clades, with clonal transmission identified both within and across health care facilities, but the reasons for this simultaneous emergence are not known [10,12,13].

The widespread use of antifungal drugs has been suggested as a contributory cause in the emergence of *C. auris* [14]. One other hypothesis is the rising ambient temperatures (caused by human activities) that might have selected thermotolerant yeasts in wetlands; and that *C. auris* might have spread through different ecosystems and hosts, such as avian birds. The tolerance to high salinity levels and ability to grow at 42 °C are points that strengthen this latter hypothesis [15,16,17]. 

Increasing number of travels to foreigner countries and exposure to healthcare in countries with extensive *C. auris* transmission as well as higher flow of migrants are factors that can contribute to the quick dissemination of this species. Further, a higher frequency higher frequency of *C. auris* was found in public hospitals in comparison to the private ones, which may be connected to overcrowding and compromised infection control measures [14].

Increasing rates of antifungal usage at a global level are also pointed out as other potential reason. Initial epidemiologic characterization of *C. auris* cases found that many patients with *C. auris* infection had been receiving antifungal drugs at the time *C. auris* was isolated, suggesting that drug pressure could have resulted in emergence of this resistant organism, at least within healthcare settings [10].

Multidrug-resistant *C. auris* may also find a reservoir in facilities such as skilled nursing units for ventilated patients and long-term acute care hospitals. Hospitals are discharging high-acuity patients to these other facilities, which do not have a comparable level of infection prevention education and staffing, with possible easier transmission to other patients.

The main reasons for the high impact of this *Candida* species are: 

(a) Most *C. auris* isolates are resistant to fluconazole. However, and contrarily to other fluconazole resistant *Candida* species, in *C. auris* resistance to other antifungal agents has also been reported, and some isolates are described as multiresistant, since they are resistant to the three classes of antifungal agents available (azoles, polyenes and echinocandins). Also, *C. auris* has higher potential to disseminate than other *Candida* species.

(b) *C. auris* appears to survive on surfaces for long periods of time and to have high ability for transmission among patients in a hospital environment, possibly associated with environmental contamination or transitory colonization of medical personnel, visitors or medical devices [18].

(c) It is a species of difficult identification since it can be confused with other *Candida* species. Their correct identification is made through biochemical or mass spectrometry methods, whose devices already have their databases updated, that is, they already contain this species’ profile. Confirmation of species identification should be done by sequencing specific DNA regions or specific PCR.

### 2.2. Which Is the Global Burden of These Fungal Infections?

**Figure 1:**
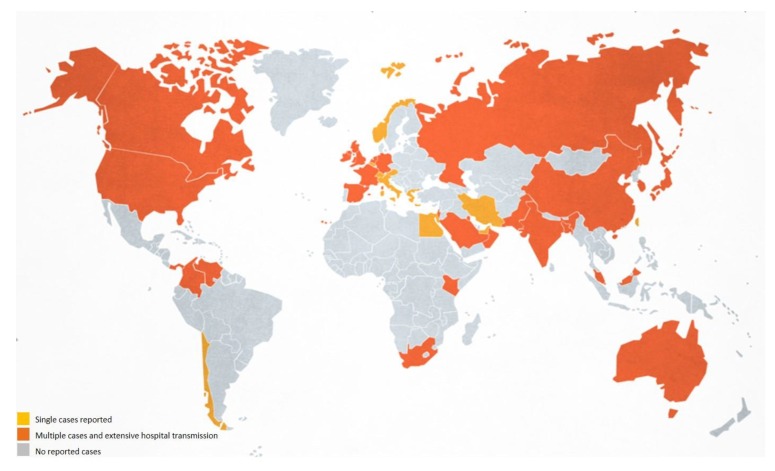
Global epidemiology of *Candida auris* for November 2019 (image adapted from CDC [19]). Single cases were reported from Austria, Belgium, Chile, Italy, Greece, Egypt, Iran, Norway, Switzerland, Taiwan and United Arab Emirates. Multiple cases and extensive hospital transmission were reported from Australia, Bangladesh, Canada, China, Colombia, France, Germany, India, Israel, Japan, Kenya, Kuwait, Malaysia, Paskistan, Netherlands, Oman, Panama, Russia, Saudi Arabia, Singapore, South Africa, South Korea, Spain, UK, USA and Venezuela [20,21,22,23,24,25,26,27,28,29,30,31,32,33,34,35].

There are no worldwide estimates of the number of *C. auris* infection/colonization cases. It is now reported in more than 35 countries, in all continents except Antarctica (Figure 1) [19]. *C. auris* has rapidly emerged as a major cause of candidemia worldwide, surpassing the number of cases caused by *C. glabrata*, *C. tropicalis*, and *Pichia kudriavzevii* in South Africa [20]. Reports of the first cases of infections/colonizations in different countries have been widely described [21,22,23,24,25,26,27,28,29,30,31,32,33,34]. In some of these countries, extensive transmission of *C. auris* has been documented in more than one hospital [35]. 

In the United States, *C. auris* was made nationally notifiable in 2018. Until March 2019, 613 confirmed cases and 1123 colonized patients have been reported in eight states [19]. The majority of the reported infections involved critically ill patients [14]. 

In Europe, from 2013 to 2017, a total of 620 *C. auris* cases were reported from six countries to European Centre for Disease Prevention and Control (ECDC). This number arose during an online survey that included reports from 29 of 30 EU/EEA countries. Cases were reported from Spain (*n*  =  388), United Kingdom (*n*  =  221), Germany (*n*  =  7), France (*n*  =  2), Belgium (*n*  =  1) and Norway (*n*  =  1). Regarding the other countries, nor colonisation or invasive infection had been detected (in 15 countries) and in seven other countries no information were made available at the national level by the National Focal Points [36].

### 2.3. Why Is C. auris Is Reported as Rapidly Spreading Worldwide as an Agent of Hospital-Associated Outbreaks?

According to recent studies, the worldwide *C. auris* population consists of distinct clades that harbour nearly identical strains within each clade (East Asia, South Asia, Africa, and South America) [11,13]. Recently, an isolate from Iran was found to be genetically distinct from the four existing clades, having a difference of >200,000 single-nucleotide polymorphisms compared with the other four clades and may constitute the fifth clade [13]. The reasons for the simultaneous emergence of this species in different geographic areas are not known. Hypotheses have included possible animal reservoirs, as well as environmental changes that may have led to this situation [10].

Whole genome sequencing (WGS) was used to show that *C. auris* isolates from four Indian hospitals were highly related and their findings were suggestive of clonal transmission [37]. Similar results were reported for Pakistan, South Africa, Venezuela, and Colombia, where *C auris* isolates within each country were highly related. However, large genetic differences were observed between isolates in different global regions, indicating that *C. auris* has substantial phylogeographical structure. Whole-genome sequencing identified a difference of 40,000–140,000 single nucleotide polymorphisms (SNPs) between *C. auris* isolates from the different regions (East Asia, South Asia, Africa, and South America) [11]. The cases detected in the USA did not represent a new clade, but they were genetically linked to the previously defined geographical clades, which suggested several introductions of *C. auris* into the US health-care system in different instances [38]. The same was observed in a European hospital using amplified fragment length polymorphism (AFLP) to analyse *C. auris* isolates from an outbreak in a cardio-thoracic center located in the United Kingdom with other isolates from other countries in order to identify a geographic origin of the ongoing outbreak [39]. AFLP analysis suggested that the London isolates formed a distinct cluster compared to other global isolates but WGS analysis showed placed the United Kingdom outbreak in the India/Pakistan clade, demonstrating an Asian origin. When all isolates from the outbreak were compared, there was a weak support for the monophyletic status of isolates within the hospital, suggesting that multiple introductions of *C. auris* occurred as well [40].

*C. auris* genome is also very different from other *Candida* species, which may explain its unique features in regards dissemination pattern, susceptibility, virulence attributes, among others. A phylogenetic analysis integrating the genomes of *C. auris*, *C. haemulonii*, *C. duobushaemulonii*, and *C. pseudohaemulonii* with other *Candida* species was performed. Comparing the genomes of these four emerging species to those of other *Candida* species, genes linked to drug resistance and virulence were identified, including expanded families of transporters and lipases, as well as mutations and copy number variants in ERG11 [41]. Gene expression analysis identified transporters and metabolic regulators specific to *C. auris* and those conserved with related species.

### 2.4. C. auris Has Been Identified as Agent of Hospital-Associated Infections. Are There Community Acquired C. auris Infections?

A report of screening on admission to the hospital (potentially reflecting burden of *C. auris* in the community) in the United Kingdom found that just one in over 2200 of admitted patients were positive for *C. auris*. However, this screening was performed in a low prevalence country, and data about the patient’s prior healthcare exposures was not available, limiting interpretation about whether *C. auris* colonization was acquired in the community or in the healthcare setting [39]. A smaller admission screening program at a trauma intensive care unit in India, a country with more documented transmission, did not find any patients with *C. auris* colonization at admission [42]. In the United States, nearly all patients have had recent healthcare exposure [10].

*C. auris* invasive infection and colonization have been identified almost exclusively in patients in high-dependency areas with the highest degree of medical intervention. Although transmission of *C. auris* in healthcare settings has been well documented, less is known about transmission in the community [10]. The possibility of this yeast also spreading into the community, farms and general environment should not be lost sight of [43]. An animal or environmental reservoir for *C. auris* has not yet been identified. Only a small proportion of cases being community-acquired is evocative of the high frequency and predilection for nosocomial transmission [44]. This suggests that a community reservoir may be unlikely; transmission to contacts in the healthcare settings is a significant risk that may depend on colonization density [44]. The role of health care workers in spreading *C. auris* is still unknown. However, during investigation of the outbreak in the United Kingdom, *C. auris* was isolated from the nares of a nurse who was providing care for a patient who was heavily colonized [39]. Moreover, an outbreak investigation in Colombia isolated *C. auris* from the hands of two health care workers and the groin of one out of six health care workers. Whole-genome sequencing established that these were genetically identical to strains isolated from a patient and surround environment [45].

The presence of *C. auris* in a non-hospital settings has been previously described in a study on potentially pathogenic fungi in swimming pools in the Netherlands and found out three *C. auris* isolates in the water of two different swimming pools, which indicates a wider presence of *C. auris*, not restricted to hospital environment [46]. However, much more data are needed to understand the ecological niche of this species and ccommunity-based studies are necessary to understand the risk of transmission outside healthcare settings.

### 2.5. What Are the Recommended Infections Control Practices in Healthcare Facilities for the Prevention of C. auris Spreading?

For all forms of candidiasis, including the mucocutaneous ones, CDC recommended standard precautions of infection control for preventing transmission in health care setting.

However, like in other microorganisms, the infection control practices are not specific to one genus or species but dependent on other factors like contagiousness/spreading ability, pathogenicity or virulence (high morbidity and mortality in the general population or in patients with particular susceptibilities), difficult treatment (multi- or pan-resistence, innate or acquired) or because they are emerging microorganisms [47,48]. *C. auris* is an emerging microorganism, with proven high ability to spread and multidrug resistance that makes it an epidemiologically important microorganism that in addition to basic infection control measures requires the implementation of contact precautions of infection control [49,50].

Detection of even a single case of *C. auris* should trigger an epidemiological investigation of an outbreak [51]. This requires, as has already been stated, that microbiology laboratories could identify precisely *C. auris* and further implementation of lab-based warning systems. In the presence of a positive case, it is necessary to determine the magnitude and severity of the problem, advise key practitioners, notify the competent authorities and management board of the institution, set up an outbreak management team, and develop a strategy plan for dissemination control with respective monitoring. Several recommendations, listed in Table 1, have been published by the Centers for Disease Control and Prevention [51], the European Center for Disease Control [52], Public Health England [53] and National Institute for communicable diseases [54].

Usually, outbreaks follow an exponential increase in the number of affected patients. Patients potentially or already colonized should be placed in single rooms with contact isolation precautions. Hand hygiene (with alcohol or chlorhexidine hand rubs), wearing of protective clothing, and skin and environmental/equipment decontamination should be performed to prevent ongoing transmission.

It is advisable to implement stringent infection control measures for all positive *C. auris* patients, including strict isolation in a single-patient room or cohorting for the duration of their hospital stay. Screening should be applied for contacts and patients previously hospitalized in healthcare settings where *C. auris* isolation was confirmed. It is mandatory to trace contacts with the aim to achieve early identification and screening of possible colonized patients that might be responsible for persistence of *C. auris* [55]. It is necessary also to form cohorts with their direct patient contacts and to perform extensive screening of them (look for the yeast presence in clinical and screening samples as nose, axilla, groin, throat, rectum or faeces, vascular line exit sites, urine, wounds, drains and respiratory specimens), as well as ceasing new admissions in the affected areas. If the outbreak persists despite all measures implemented, then it will be necessary to include professionals in the screening [51,56].

Healthcare personnel caring for patients with *C. auris* must faultlessly adhere to the five moments of hand hygiene (before touching a patient, before clean/aseptic procedures, after body fluid exposure/risk, after touching a patient, and after touching patient surroundings). This is the single most important practice to reduce the transmission of infectious agents in healthcare settings, and use adequate Personal Protective Equipment (wear a gown and gloves) in all interactions that may involve contact with the patient or the patient’s environment [39,57]. For now, CDC or ECDC do not advocate a decolonization protocol or change the antiseptic alcohol-based solution used in hand hygiene. Skin and environmental/equipment decontamination should be performed to prevent ongoing transmission [55]. Survival in the ICU environment appeared to facilitate the persistence and transmission of this organism and lead to the vicious cycle of colonization-spread-infection, very difficult to control. Reusable patient equipment may serve as a source of health care-associated outbreaks of infection with *C. auris* [7]. Thus, it is very important to have a cleaning protocol and strengthen the regular decontamination of the equipment and environment, especially high-touch areas with a chlorine-based product. For terminal cleaning/disinfection a ‘no-touch’ automated room disinfection system must be considered (as hydrogen peroxide or UV-C) to eliminate *C. auris* transmission [56,58].

### 2.6. Why Is C. auris Considered an Emerging Multidrug-Resistant (MDR) Yeast? Are There Other Attributes in C. auris Different from Other Candida Species?

While distantly related to *C. albicans* and *C. glabrata*, *C. auris* is closely related to rarely observed and often multidrug-resistant species from the *C. haemulonii* clade. *C. auris* and their phylogenetically related species *C. haemulonii*, *C. duobushaemulonii*, and *C. pseudohaemulonii* represent an emerging clade of invasive fungal pathogens, all displaying a multidrug-resistant (MDR) profile, most commonly to amphotericin B and also reduced susceptibility to azoles and echinocandins [59]. A high proportion of *C. auris* isolates are resistant to more than one class of antifungal agents (azoles, echinocandins and polyens). This indicates that probably more than one molecular mechanisms are underlying this resistance and this is currently under investigation. Efflux pump activity contributes to azole resistance in other *Candida* species and recent studies suggest their importance in *C. auris* as well [60,61,62].

Most of the genes associated with drug resistance and pathogenesis in *C. albicans* are conserved in *C. auris*, *C. haemulonii*, *C. duobushaemulonii*, and *C. pseudohaemulonii*. Orthologs genes noted to confer drug resistance in *C. albicans* were identified in *C. auris*; this resistance occurs either by acquiring point mutations, increasing transcription, or copy number variation [41,60]. 

Recently, it was shown that older *C. auris* cells presented enhanced fluconazole associated with increased rhodamine 6G (R6G) efflux [63]. The higher efflux in the older cells correlated with overexpression of the efflux pump encoding gene *CDR1* (fourfold). On the other hand, Hsp90 enables tolerance of clinical isolates to azoles [64]. However, high-level azole resistance was independent of Hsp90, but dependent on the ABC transporter *CDR1*, deletion of which resulted in abrogated resistance. Inhibition of Hsp90 blocks calcineurin-dependent stress responses and cell wall integrity signalling, thereby reducing antifungal tolerance of clinical isolates and transforming azole activity from fungistatic to fungicidal [64].

The annotated genome assemblies of *C. auris*, *C. haemulonii*, *C. duobushaemulonii*, and *C. pseudohaemulonii* contain a single copy of the *ERG11* azole target and the *UPC2* transcription factor that regulates the expression of genes in the ergosterol pathway, as well as all the gene components of the ergosterol biosynthesis pathway [41].

An eightfold upregulation of the azole target encoding gene *ERG11* was noted in *C. auris* older cells. Analysis of genomic DNA from older cells by qPCR indicates that transient gene duplication of *CDR1* and *ERG11* causes the observed age-dependent enhanced fluconazole tolerance [63]. Twelve ERG11 mutations, which have been found in fluconazole-resistant, but not wild-type *C. albicans*, have been found in *C. auris* in silico analysis. Three of these mutations have been directly linked to drug resistance in *C. albicans*, suggesting that they contribute to the resistance observed in *C. auris* as well [10].

Furthermore, detection of azole resistant mutations by comparing Erg11 amino acid sequences between *C. albicans* and *C. auris* showed that alterations at azole-resistance codons in *C. albicans* were present in *C. auris* isolates. These substitutions were strongly associated with country-wise-specific geographic clades [11]. Resistance is probably inducible under antifungal pressure, resulting in rapid mutational changes [64].

Elevated echinocandin minimal effective concentrations (MECs) are likely the result of FKS mutations observed in *C. auris* isolates, such as the S639F mutation observed in isolates from India [65]. These mutations correspond to known mutations in other *Candida* species, which have been directly linked to echinocandin resistance [66].

Resistance to amphotericin B in *C. auris* is not well understood yet but whole-genome sequencing of resistant isolates identified four novel nonsynonymous mutations, highlighting a potential association with resistance. These mutations included those in genes with homology a transcription factors of *C. albicans* and a membrane transporter [45]. 

### 2.7. Which Factors May Contribute to the High Mortality Associated to C. auris Infections?

Patients infected or colonized with *C. auris* present frequently several underlying health conditions or comorbidities (diabetes, chronic/acute kidney failure/pathologies, immunosuppression conditions, solid tumour/malignancies, cardiovascular diseases, and liver diseases) [43]. These patients have often undergone multiple medical interventions, including surgical procedures, mechanical ventilation, vascular catheterization, and gastrostomy tube placement [11,49]. The most important predisposing factors to *Candida* infections are iatrogenic. Of these, probably the most significant is the exposure to broad-spectrum antibiotics for treatment and prophylaxis of several infectious conditions.

The pathogenicity of *C. auris* was explored in an invertebrate moth larvae (*Galleria mellonela*) model of invasive candidiasis, and found that some *C. auris* strains to be considerably more virulent than other no filamentous *Candida* isolates, and to a level comparable to that of *C. albicans* [67]. Contrarily to *C. albicans*, where morphological transition from nonpathogenic yeast form to pathogenic hyphae form has been identified as initial step of pathogenesis, no morphological transition has been observed in *C. auris* isolates.

The morphology of *C. auris* on the rich media YPD and YPD plus 10% NaCl has a round appearance on the former medium, but an elongated shape on the latter. Interestingly, a small portion of highly elongated and pseudohyphae-like forms were observed when grown on YPD plus 10% NaCl [68]. These results suggest that high-salt stress may result in incomplete cell division, leading to the formation of pseudohyphae-like forms. *C. auris* fails to form chlamydospores after growth on cornmeal agar when incubated for 3 days at 30 °C and does not germinate when incubated with fetal bovine serum. *C. auris* undergoes a morphogenetic transition from yeast to filamentous growth in response to HSP90 depletion or cell cycle arrest but not in response to other cues that induce *C. albicans* filamentation [64]. Recently, a novel phenotypic switching system in *C. auris* has been reported which transits cells in three different cell types—typical yeast, filamentation-competent yeast and filamentous cells [69]. In addition to its role in enabling the emergence and maintenance of azole resistance, Hsp90 governs temperature-dependent morphogenesis in *C. albicans*. The ability of *C. albicans* to transition between yeast and filamentous forms is a key virulence trait that is triggered by a wide variety of environmental cues [70]. However, exposure to various cues that induce *C. albicans* filamentation does not induce morphogenesis in *C. auris*, suggesting alternative pathways regarding the yeast-to-filament transition between the two *Candida* species [71]. Higher levels of salt and higher temperatures seem to be the ideal conditions for *C. auris* development [32]. 

*C. auris* has been described as highly virulent also due to its ability to form biofilms [46]. *Candida* species are adapted to surface adherence and biofilms formation in body surfaces as well prosthetic devices, including urinary catheters and intravascular devices [72]. Biofilm formation is a strain-dependent trait in *C. auris,* strongly associated with the type and phenotypic behaviour of the isolates. Aggregative and nonaggregative phenotypes were found to be predominantly associated with colonising and clinical isolates of *C. auris,* respectively [73]. Furthermore, multiple transporter genes and protein kinases, which may facilitate the acquisition of drug resistance, have been identified in *C. auris* [51]. *C. auris* biofilms did not show susceptibility to any antifungal agent, showing minimal biofilm eradication concentration (MBECs) that were up to 512-fold higher than the minimal inhibitory concentrations (MICs) [74].

How biofilm formation by *C. auris* may influence immunity is unknown, however biofilms formed by other *Candida* species resist phagocytic killing [75,76]. All arms of the immune system are involved in the response of *Candida* infections. Lymphocytes are crucial to the development of cell-mediated immunity, and monocytes and neutrophils damage and destroy pseudohyphae and blastospores. Deficiencies in T-helper 17 cell line impair the mucosal immune response to *C. albicans*, and patients with significant neutrophil dysfunction or leukopenia have a strong propensity toward the development of candidemia.

The host immune response induces the release of pro-and anti-inflammatory cytokines in order to mediate the immune response [77]. The innate immune cells may suffer an enhancement on cytokine release by monocytes and macrophages in a T-cell independent manner. *Candida* species have developed evasion mechanisms that may circumvent the host immune response [78,79].

Neutropenia, a common risk factor for invasive candidiasis has not been reported for *C. auris* infection [11,80]. Human neutrophils failed to engage *C. auris*, phagocytose the yeasts, or release neutrophil extra-cellular trap (NET) formation [81]. Mice deficient in neutrophil elastase, an essential NET component, survived to high-dose *C. auris* intravenous challenge [82]. 

Little is known regarding the mechanisms underlying this phenomenon or how the genetic diversity of *C. auris* may influence evasion of neutrophil engagement. In contrast to *C. albicans*, similar pattern of phagocyte evasion has been observed for *C. lusitaniae*, a species phylogenetically close to *C. auris* [83]. This prompts the question of an altered fungal component that is shared for the two species, but divergent from more distantly related *Candida* species [84]. Peripheral blood mononuclear cells appear to elicit unique cytokine responses encounter with *C. auris* and *C. albicans*, suggesting exposure to different fungal components [85]. The observation that neutrophil exhibit reduced activity against *C. auris* may contribute to poor outcome for patients with invasive disease even for patients treated with appropriate antifungals [11,84].

Despite the results obtained regarding the possible effect of NETs, in the same study the authors also used inbred mouse strains highly susceptible for *C. auris* invasive infection. This A/J mouse strain carries a loss-of-function mutation in the structural gene for the C5 component of the complement pathway, that plays several critical roles in the host response to infection, including target lysis and phagocyte recruitment [82]. 

*C. auris* infections are currently associated with candidemia, high mortalities, persistent fungemia and therapeutic failure at risk patients, many of whom have multi comorbidities. The mortality rates have varied significantly among geographic regions, with risk factors ranging from the presence of invasive devices to immunocompromised conditions [43,86]. Mortality rates from initial studies were concerning, although *C. auris*—attributable mortality cannot be established from those studies. As such, the overall attributable mortality rates are unclear. The number of deaths attributable to candidemia, as opposed to underlying medical conditions, may be difficult to quantify [62]. The significance of *C. auris* as a human pathogen remains unclear.

### 2.8. Does C. auris Isolated from Sites Other Than the Blood Stream, such as Urine and Respiratory Tract, Represent Infections or Colonizations?

*C. auris* seems to have a special predilection for the skin. Data from swabs taken to assess patients for *C. auris* colonization show that the axilla and groin are the highest yield sites to detect colonization, followed by nares [10,21]. Specimens from urine, stool, vagina, and rectum have also yielded *C. auris*. Despite the majority of *Candida* species can cause oral or esophageal disease, *C. auris* is not frequently reported at these sites. The activity of a salivary cationic peptide, histatin 5, against *C. auris* was examined and the vast majority of isolates were found to be highly sensitive to this peptide, particularly those exhibiting antifungal resistance, which may explain the low frequency of *C. auris* isolation in those sites [87]. Patients with clinical infection with *C. auris* were typically found to be colonized in noninvasive sites, like skin, long after resolution of invasive infection. Patients may become colonized with *C. auris* without active infection. Although asymptomatic colonization with *C. auris* does not require antifungal treatment, it is important to identify individuals who are colonized [10]. Previous colonization can lead to invasive infections as well as to outbreaks. Patients who were colonized with *C. auris* can develop an invasive bloodstream infection days to months after becoming colonized [10]. Candidemia usually occurs after an event, such as the placement of a new line or tube, which provides the opportunity to introduce the organism from the skin into the bloodstream.

In a study of a *C. auris* outbreak in an intensive care setting, the risk for colonization or infection was associated with exposure to axillary temperature monitoring [7]. Whole-genome sequencing found that the patient and probe isolates were all highly related.

### 2.9. How Can the Clinical Presentation of an Infection Caused by C. auris Be Distinguished from That by Other Candida Species?

The incidence of *C. auris* infections is significantly higher in patients with primary or acquired immunosuppression, secondary to therapeutic management of hematologic malignancies, bone marrow transplantation, and other condition requiring immunosuppressive agents [18,39]. A donor-derive transmission of *C. auris* during lung transplantation was also described [88]. Nevertheless, neutropenia is a common risk factor for invasive candidiasis that has not been reported for *C. auris* infection [11,80]. The unexpected fitness of *C. auris* in several immunocompetent animal models suggests that this species may withstand host immune responses that are typically sufficient to prevent invasive disease caused by other *Candida* species [82].

*C. auris* has been documented to cause infections in patients of all ages. In most cases, clinical presentation is nonspecific and it is often difficult to differentiate between other types of systemic infections. Most of the reported cases were isolated from blood and other deep-seated sites of infection (including invasive devices and catheters tips) [43].

Isolations from nonsterile body sites (lungs, urinary tract, skin and soft tissue, and genital apparatus) may more likely represent colonization rather than infection. As for other *Candida* species, the presence of signs and symptoms of infections of the site where *C. auris* has been isolated from can help to differentiate between simple colonization and infection [89].

### 2.10. How Does First-Line Therapy for C. auris Infections Differ from Therapy for Other Candida Species?

The choosing of initial antifungal therapy to an invasive candidiasis, depends of several important factors, such as the source of infection, severity of the illness, evidence to suggest involvement of central nervous system or cardiac valves, presence of including central venous catheter and other intravascular devices, comorbidities, underlying disorders, especially immunosuppression, and the susceptibility patterns of *Candida* species.

More than 95% of invasive disease is caused by the most commons pathogens *C. albicans*, *C. glabrata*, *C. tropicalis*, *C. parapsilosis*, and *P. kudriavzevii*. According to data from the United States, *C. albicans* has a low incidence of fluconazole resistance, approximately 0.5%–2%. *C. tropicalis*, *C. parapsilosis*, and *C. glabrata*, on the other hand, have higher rates at 4%–9%, 2%–6%, and 11%–13%, respectively. The emerging yeast *C. auris* can exhibit a rate of resistance to fluconazole as high as 93% [4].

Broadly speaking, the pharmacological treatment of candidemia comprises three antifungal groups: amphotericin B-based preparations, azoles, and echinocandins. Fluconazole is the drug of choice as initial treatment in most cases of candidemia, in stable patients without previous intake of azoles. Lipid formulation amphotericin B is a good choice, in patients with proven intolerance, and limited availability, to other antifungal agents. The echinocandins demonstrate significant fungicidal activity against most *Candida* species [90]. However, these agents are only available as parenteral preparations. Despite this limitation, the efficacy, few drug interactions, and concerns about fluconazole resistance have led echinocandin as recommended initial therapy for candidemia in non-neutropenic or neutropenic patients. The change in the treatment scheme from echinocandin to fluconazole is recommended after a 5–7 days period, in patients whose *Candida* species is sensitive to fluconazole, and are clinically stable [89]

Although susceptible *C. auris* strains, specifically to fluconazole, have been described, most *C. auris* strains have been reported to be high resistant to fluconazole and/or to other azoles, and to amphotericin B, with a minority being resistant to 5-flucytosine and the echinocandins [43]. Almost half of isolates are multidrug-resistant (resistant to ≥ 2 antifungal classes), and a low number (4%) exhibit resistance to all classes of antifungals [11,14,91]. Higher resistance to fluconazole, in a *Candida* non-*albicans* species, has become one of the distinguishing characteristics of a potential *C. auris* infection [51]. Efflux pumps, mutations in the ERG and FKS genes, and biofilm formations are potential *C. auris* resistance mechanisms [41,92,93].

*C. auris* invasive infections represent a therapeutic challenge, and no consensus exists for optima treatment. Based on the most frequent resistance profiles, micafungin is the recommended first-line treatment for most *C. auris* infections in adults; however, this treatment is expensive and is not easily available in countries with more limited resources. Patients should be monitored closely for resolution of infection, given micafungin resistance has developed in patients after exposure to the drug. Switching to, or adding, liposomal amphotericin B or isavuconazole could be considered, when no clinical response or persistent fungemia for > 5 days is observed [94,95]. For neonates and infants under 2 months of age, amphotericin B is recommended as the first line treatment, and echinocandin should only be considered in rare circumstances and only after checking that the central nervous system has not been affected [96]. Due to echinocandins limited penetration into the central nervous system, other antifungal agents such as flucytosine, posaconazole, and isavuconazole could be used, depending the isolate in vitro susceptibility.

Candidemia management includes not only early antifungal therapy but also a wide range of measures, such as drainage of collections, central venous catheter or other devices removal, and if blood cultures remain positive, then search for a metastatic focus, such as an abscess or endocarditis [89]. The duration of antifungal treatment in candidemia is generally determined by the individual clinical and mycological response to therapy. It should be continued at least for two weeks after blood cultures become negative [89]. The treatment of colonization without evidence of active infection is strongly discouraged [12].

There is an urgent need to expand the antifungal armamentarium, especially in the face of rising incidence and continuous spread of multidrug resistant isolates of *C. auris*. Among some newly investigated compounds, SCY-078, a novel triterpene glucan synthase inhibitor, has shown promising in vitro results against *C. auris* [97]. 

### 2.11. Which Are the Most Recent Tools Available for C. auris Detection and Identification?

CDC advises *Candida* identification to species-level in the following situations: (a) when clinically indicated in the care of a patient; (b) when a case of *C. auris* infection or colonization has been detected in a facility or unit; (c) when a patient has had an overnight stay in a health care facility in the previous 6 months in a country with *C. auris* transmission. Laboratories should also review past records to identify confirmed or suspected cases, as well as conduct prospective surveillance [98]. Because contact precautions and other infection control measures are not recommended for most other *Candida* species but are essential for the control of *C. auris*, species identification is important not only for infection control but also for treatment [99].

As previously referred, diagnostic laboratories using standard biochemical identification can only identify *C. auris* if the system contains a reference in its database. As databases are being updated, accurate identification becomes more possible. Even so, misidentifications of strains from certain *C. auris* clades have been reported and, for example, all VITEK identifications of *C. duobushaemulonii* should be forwarded for further identification [100]. In addition, multidrug resistant *Candida* isolates should also be precisely identified. Thus, laboratories can reliably identify an isolate by sequencing the D1–D2 region of the 28s rDNA or the internal transcribed spacer (ITS) regions of rDNA, *C. auris* specific PCR/qPCR or matrix assisted laser desorption ionization-time of flight mass spectrometry (MALDI-TOF MS) performed directly from the culture may be used as confirmatory identification methods [101,102,103,104,105,106,107].

Regarding DNA detection directly from the biologic product, several molecular-based assays have been recently developed, from conventional PCR through real-time PCR, to more complex T2 magnetic resonance or Loop-mediated Isothermal Amplification (LAMP) [107,108,109,110]. Most of the developed assays (commercial or in-house) are characterized by their high sensitivity and specificity.

Molecular methods are also being applied to detect resistance in *C. auris* using allele-specific molecular beacons and DNA melting curve analysis following asymmetric PCR. A duplex ERG11 assay and a simplex FKS1 HS1 assay were developed to identify the most prominent resistance-associated mutations concerning resistance to azoles and echinocandins, respectively [111]. 

### 2.12. What Do We Need to Study to Advance Our Understanding of C. auris?—Future Perspectives 

The implementation of antimicrobial stewardship programs is warranted to minimize unnecessary antibiotic use. Stewardship teams can also assist with rapid case identification, appropriate management, and coordinate with infection prevention to minimize transmission [12].

There are multiple unanswered questions regarding *C. auris*, population prevalence, and environmental contamination, effectiveness of infection prevention and control, and impact on patient mortality [21,112]. In order to understand how it spreads and discover possible reservoirs, it is essential to know the ecology, the natural environment, and distribution of this species. It is also important to explore possible reasons to this recent emergence, namely the environmental presence of azoles or the potential effect of climate change, as global warming and UV radiation, on this sudden emergence [15,16,17]. Community-based studies will help clarify whether *C. auris* is associated mainly to hospital infections or whether there is widespread hospital and community carriage [21]. 

Regarding the microorganism itself, more whole genome sequencing studies are needed to unveil mechanisms of resistance and virulence attributes and those discoveries must be validated with in vitro and in vivo models. Furthermore, serial studies on antifungal susceptibility should be done in order to establish clinical breakpoint to the different antifungals. 

Despite what is already known, much more has yet to be explored in *C. auris*. Scientific and clinical communities around the world must continue to develop research project towards the discovery of the ecological niche of this organism, a better understanding of specific genes associated to virulence that could be used as antifungal targets and the study of host genetic markers that can be associated with a worse outcome.

## Figures and Tables

**Table 1:** Recommendations from the different institutions regarding infection control practices in *C. auris.*

Institutions	Recommendations for *C. auris* Infection Control Practices
**Public Health England**	Isolation of all patients colonised or infected with the organism in a single room, ideally with ensuite facilities, wherever possible, side rooms or cohorted
**Centre for Opportunistic, Tropical and Hospital**	Minimization of the number of staff who care for the *C. auris* patient. If multiple *C. auris* patients are present in a facility, consider cohorting staff that care for these patients.
**European Center for Disease Control**	Equipment used for the infected/colonised patient should not be shared with other patients on the ward unless between-patient decontamination can be assured.
**Center for Disease Control and Prevention**	Strict adherence of healthcare workers to standard precautions including hand hygiene using soap and water followed by alcohol hand rub on dry hands
	Personal protective equipment in the form of gloves and aprons
	Affected patients, visitors and family members should be briefed about the importance of hand hygiene and visitors encouraged to use protective aprons
	Single-patient use items such as blood pressure cuffs and pillows should be considered
	If a patient needs to be taken out of the side room or bay to theatre, procedure room, or for imaging, they should be scheduled last on the list for the day and the environment cleaned
	Hypochlorite is currently recommended for cleaning of the environment at 1000 ppm of available chlorine
	CDC recommends use of an Environmental Protection Agency (EPA)-registered hospital-grade disinfectant effective against *Clostridioides difficile* spores
	If any noncontact disinfection is used (e.g., gaseous hydrogen peroxide or UV), full cleaning and disinfection preceding it should still occur
	Routine screening for *C. auris* at the time of hospital admission is not currently recommended
	Periodic reassessments for presence of *C. auris* colonization (e.g., every 3 months) for a patient with known *C. auris* colonization could help inform duration of infection control measures
	“Flag” the patient’s record to institute recommended infection control measures in case of re-admission
